# Effect of Pore Filling on Properties of Nanocomposites LiClO_4_–MIL–101(Cr) with High Ionic Conductivity

**DOI:** 10.3390/nano12193263

**Published:** 2022-09-20

**Authors:** Nikolai Uvarov, Artem Ulihin, Valentina Ponomareva, Konstantin Kovalenko, Vladimir Fedin

**Affiliations:** 1 Institute of Solid State Chemistry and Mechanochemistry, SB RAS, Kutateladze 18, Novosibirsk 630090, Russia; 2 Nikolaev Institute of Inorganic Chemistry, SB RAS, Acad. Lavrentiev Ave. 3, Novosibirsk 630090, Russia

**Keywords:** nanocomposite solid electrolytes, metal-organic frameworks, MIL-101(Cr), high lithium ion conductivity, model of composite, calculation of conductivity

## Abstract

Experimental data on nitrogen adsorption, pellets density and ionic conductivity of nanocomposite solid electrolytes (1−x)LiClO_4_–xMIL-101(Cr) were interpreted in frames of the model of the composite in which the lithium salt fills the pores of a metal-organic framework MIL-101(Cr). According to the model, the concentration of lithium salt located in the pores reaches a maximum at the concentration *x* = *x*_max_ which is defined by a ratio of the molar volume of LiClO_4_ and the total volume of accessible pores in the MIL-101(Cr) framework. The model allows one to describe the dependences of pore volume and pellet density on the concentration of MIL-101(Cr). Conductivity of the composites were successfully described by two separate mixing equations for concentration ranges *x* < *x*_max_ and *x* > *x*_max_. In the first concentration region *x* < *x*_max_, the composite may be regarded as a mixture of LiClO_4_ and MIL-101(Cr) with completely filled pores accessible for LiClO_4_. At *x* > *x*_max_, the total amount of lithium perchlorate is located in the pores of MIL-101(Cr) and occupies only part of the volume of the accessible pores. It was found that *x*_max_ value determined from the concentration dependence of conductivity (*x*_max_ = 0.06) is noticeably lower than the corresponding value estimated from adsorption data (*x*_max_ = 0.085) indicating a practically complete filling the pores of MIL-101(Cr) in the composite pellets heated before conductivity measurements.

## 1. Introduction

Lithium batteries are attractive devices for electrical energy storage due to a high theoretical energy density. Among them, solid state lithium-ion batteries are of special interest in which solid electrolytes with unipolar lithium-ion conductivity can be used as gas-tight lithium ion permeable membranes. To date, many lithium ion-conducting solid electrolytes are known. There are reviews devoted to such systems [1,2,3,4,5,6]. Mostly, these electrolytes are ceramic and can be prepared using solid state synthesis at elevated temperatures. Ceramic electrolytes have high strength, but they are brittle and often not mechanically compatible with solid electrodes. In this regard, composite solid electrolytes are of special interest as their physical properties may be easily adapted to a particular application by variation of the type and concentration of the components. The main reason for conductivity enhancement in the composite solid electrolyte is the interface interaction between the ionic salt and the heterogeneous additive. Such interaction can result in two main effects: the increase in the concentration of point defects in the vicinity of the interface between the ionic salt and the additive [7] and formation of new disordered, mostly amorphous, interface-stabilized states of ionic salt in the composites. The latter effect may be observed in nanocomposites with heterogeneous dopants having the particle size of the order of 10 nm or a specific surface area of order of 100 m^2^/g [8,9]. Earlier, we investigated a series of nanocomposite solid electrolytes based on LiClO_4_ [10,11,12]. It was demonstrated that the addition of nanocrystalline oxides such as Al_2_O_3_ [10], MgO [11] and SiO_2_ [13] leads to a sufficient increase in the conductivity of lithium perchlorate up to values of 10^−3^−10^−2^ S/cm at 200 °C.

Porous matrix materials with regular microstructure can be also used as heterogeneous additives for preparation of composite solid electrolytes. If the pore size is sufficiently small and the adhesion energy sufficiently high then the ionic salt will penetrate into the pores forming the nanocomposite, in which the effective grain size of the ionic salt is equal to or less than the pore size. In this case, due to a strong interface interaction, the ionic salt in the nanocomposite may transform into a metastable state possessing unusual physical or chemical properties. In our earlier work [14], it was demonstrated that LiClO_4_ inserted into MCM-41 silica mesoporous matrix (with ordered pores) is amorphous and the nanocomposite solid electrolytes LiClO_4_−MCM-41 have a high ionic conductivity.

**Figure 1:**
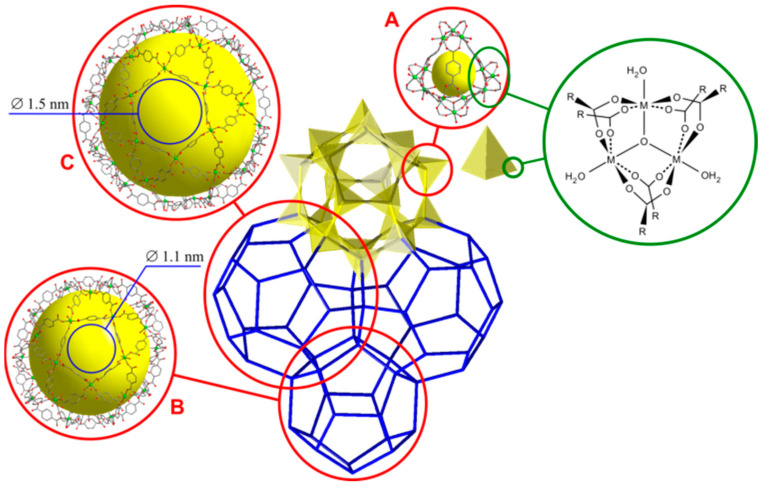
The representation of the MIL-101(Cr) zeolite-like structure formed by three-nuclear clusters [Cr_3_O(H_2_O)_3_(O_2_CR)_6_]^+^ and terephthalate linkers, which has three types of cages: microporous cages A with inner diameter of 8 Å and windows size of ca. 4 Å; small mesocages B with inner diameter of 29–30 Å and windows size of ca. 11 Å and large mesocages C with inner diameter of 34–38 Å and windows size of ca. 15 Å.

Porous matrixes based on metal-organic frameworks (MOFs) are promising systems for preparation of various functional nanocomposites [15,16,17,18,19]. Typical representatives of MOFs are chromium (III) terephthalate Cr_3_O(H_2_O)_2_(C_6_H_4_(COO)_2_)_3_X·nG, where X = OH^−^, F^−^; G are guest molecules, mainly H_2_O and other solvents, known as MIL-101(Cr). According to the results of X-ray diffraction, TG analysis and adsorption measurements, after prolonged heating in vacuum at 180 °C the compound MIL-101(Cr) has chemical composition Cr_3_O(C_6_H_4_(COO)_2_)_3_F with the molecular weight of M = 683.3 g/mol. The framework easily absorbs water with formation of a hydrated form Cr_3_O(H_2_O)_2_(C_6_H_4_(COO)_2_)_3_X·15H_2_O under ambient conditions with M = 825.47 g/mol. The crystal structure of MIL-101(Cr) is characterized by a large cubic unit cell with the parameter of 89 Å (symmetry space group Fd-3m, Z = 272) and represents a zeolite-like three-dimensional framework (Figure 1) containing ordered pores of three types with the size ranging from 8 to 38 nm with the total pore volume of 1.9 cm^3^/g [16].

It was shown that MIL-101(Cr) framework can be used for immobilization of liquid electrolytes which fill the pores and provide high conductivity of the composite obtained. The composites with such liquid electrolytes as strong acids, ionic liquids or solutions of salts were obtained and investigated [17]. The composites containing solid electrolytes in the pores of MIL-101(Cr) are less studied. It was reported that acidic salt CsHSO_4_ can be inserted into the pores of MIL-101 to form nanocomposites with high proton conductivity [20]. Results of the study of nanocomposite solid electrolytes prepared by impregnation of LiClO_4_ into the pores of the metal-organic framework MIL-101(Cr) were recently reported [21]. It was shown that at temperatures below 100 °C and relative humidity of 50% the composites easily absorb water with formation of liquid phase in the pores and have conductivity values typical for aqueous solutions. Conductivity decreases with diminishing humidity, however, and complete dehydration may be achieved only after prolonged heating in vacuum at 170 °C. The concentration dependence of the conductivity of dehydrated samples passed through a maximum and reaches 3 × 10^−3^ S/cm at 160 °C. The increase in conductivity was accompanied by the decrease in the activation energy of conductivity and the disappearance of the reflections attributed to lithium perchlorate in X-ray diffraction patterns.

Several approaches have been earlier proposed for quantitative analysis of ionic conductivity of composite solid electrolytes, such as different variants of the effective medium theory [22,23,24], percolation models [25,26] or mixing equations [27,28]. The effect of interfaces on conductivity of composites, including nanocomposites, was considered in earlier papers [23,24]. However, all these approaches are applicable to the composites comprising randomly mixed particles of insulating and conducting phases. Moreover, the expressions obtained using the effective medium theory are rather complicated. Alternatively, mixing equations allows easy fitting of experimental conductivity data for composites, including the contribution of the interfaces [27,28].

Special approaches should be elaborated for composites based on highly-porous MOF matrices taking into account the pore filling effects. To date, no papers on the quantitative description of conductivity in such systems were reported. In the present work, nanocomposite solid electrolytes LiClO_4_–MIL-101(Cr) prepared with dehydrated MIL-101(Cr) were investigated and new X-ray, conductivity and adsorption data were obtained in addition to those reported in our previous paper [21]. The model of pore filling was elaborated and applied to description of the experimental data. For a quantitative interpretation of the transport properties, a modified mixing equation was used, based on the pore filling model.

## 2. Materials and Methods

Metal-organic framework MIL-101(Cr) was synthesized as reported earlier [29]. Lithium perchlorate (chemical grade) was recrystallized in deionized water and heated at 300 °C for 2 h for complete dehydration. Prior to experiments, MIL-101(Cr) was heated at 150 °C for 2 h for removal of water adsorbed in the pores. Purified components were mixed in agate mortar under the flow of pure argon. X-ray diffraction studies were carried out with a Bruker D8 Advance diffractometer (Bruker Elemental GmbH, Kalkar, North Rhine-Westphalia, Germany) using Cu Ka radiation. To avoid fast hydration of the composites in air during the experiment, the samples were covered by protecting polyethylene film. Morphology of the initial sample MIL-101(Cr) was studied using a scanning electron microscope Hitachi S-3400N (Hitahci, Tokyo, Japan) in back-scattered electron mode. High resolution images of the samples were obtained by transmission electron microscopy using a 120 kV electron microscope JEM 1400t (Jeol ltd., Tokyo, Japan). The porous structure was analyzed using the nitrogen adsorption technique on a Quantochrome Autosorb iQ gas sorption analyzer (Quantochrome Instruments, Boynton Beach, FL, USA) at −196 °C. Initially, the compound was activated under a dynamic vacuum at 180 °C for 6 h. The nitrogen adsorption—desorption isotherms were measured within the range of relative pressures from 10^−6^ to 0.995. The specific surface area was calculated from the data obtained using the conventional BET and DFT models. For conductivity measurements, initial mixtures were compacted at 200 MPa into pellets with two thin silver electrodes pressed into upper and bottom surfaces of the pellet. All pellets were stored in a dry atmosphere. Conductivity measurement techniques were reported elsewhere [21]. On the first heating, the conductivity of the samples was relatively high, but when measured in isothermal mode, the conductivity values were not stable. In order to reach good thermal stability and reproducibility of the data, the pellets were heated at 170 °C for 12 h. Only these data will be discussed.

## 3. Results and Discussion

### 3.1. X-ray Diffraction and Morphology Studies

**Figure 2:**
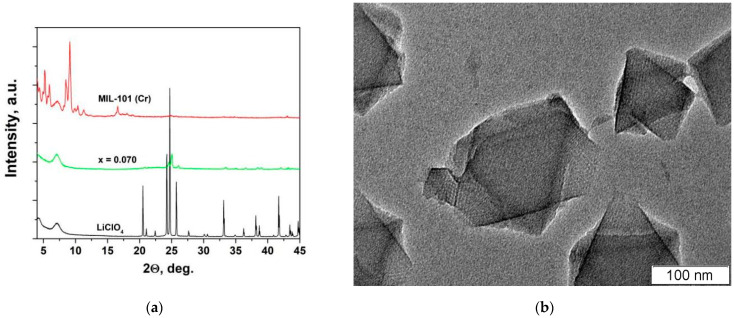
X-ray diffraction data for pure components and (1−x)LiClO_4_−xMIL-101(Cr) composite with x = 0.07 after prolonged heating at 170 °C in vacuum (**a**) and electron microscopy image of initial MIL-101(Cr) crystalline sample used for preparation of the composites (**b**).

Figure 2a shows X-ray diffraction patterns of the initial compounds and a composite (1−x)LiClO_4_–xMIL-101(Cr) with x = 0.07 (where x is the molar fraction) after prolonged heating at a temperature of 170 °C in vacuum. The diffraction pattern of pure MIL-101(Cr) corresponds to the data reported in the literature [30]; no reflections attributed to other phases are found. The X-ray diffraction pattern of pure LiClO_4_ corresponds to the pattern for the dehydrated lithium perchlorate [31]. A systematic weak shift of all reflections by 2θ = 0.04° in composites was caused by the presence of protecting film which was put on the sample surface during the experiment.

It can be seen that the diffraction peaks of both lithium perchlorate and MIL-101(Cr) are practically absent in the 0.93LiClO_4_-0.07MIL-101(Cr) composite. The decrease in the intensity of the MIL-101(Cr) peaks can be explained by insertion of the salt in the pores of MIL-101(Cr) accompanied by a strong decrease in the diffraction contrast between the pore volume and the framework structure. Such an effect was reported earlier for ionic salt MOF composites used for seasonal heat storage applications [32,33,34,35]. The absence of reflections of LiClO_4_ in the diffraction patterns can be explained by complete amorphization of the ionic salt in the nanocomposites formed as a result of the penetration of the salt into the pores. According to the results of thermal analysis, the formation of the interface-induced amorphous phase of LiClO_4_ takes place in nanocomposites of LiClO_4_ with nanocrystalline oxide additives Al_2_O_3_ or MgO at sufficiently high concentration of the additive [10,11,12]. A similar effect was also reported for nanocomposites in which ionic salt was inserted into silica mesoporous materials with ordered pores LiClO_4_-MCM-41 [14] and CaCl_2_-SBA-15 [27]. A similar effect was observed for the acid salt CsHSO_4_ imparted into the pores of MIL-101(Cr) [20]. The reason for the amorphization of the ionic salts in the nanocomposite is likely to be the size effect accompanied by a strong interface interaction between ionic salt and nanostructured additive [8,9]. Figure 2b shows an electron microscopy image of an initial MIL-101(Cr) sample. It is seen that the sample consists of well-crystallized particles 100–200 nm in size with mesopores oriented along the crystallographic axis. Unfortunately, attempts to obtain electron microscopy images of appropriate quality for the composites under study were unsuccessful due to a high hygroscopicity of the samples.

### 3.2. Pore Size Distribution in the Nanocomposites

**Figure 3:**
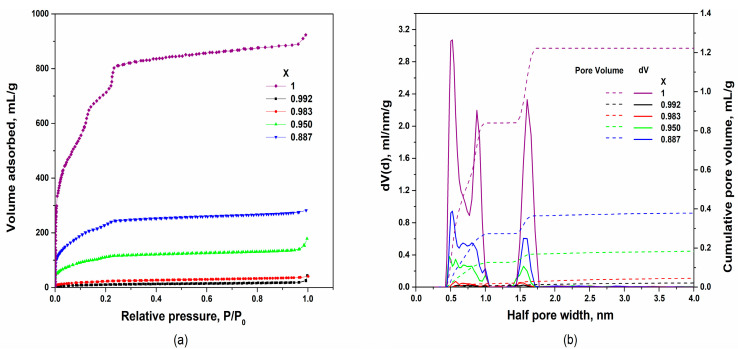
Nitrogen adsorption-desorption isotherms obtained at 77 K (**a**) and calculated DFT pore size distributions (solid lines) and cumulative pore volumes (dashed lines) for (1-x)LiClO_4_–xMIL-101(Cr) composites (**b**).

The adsorption isotherms of (1−x)LiClO_4_−xMIL-101(Cr) composites and pure MIL-101(Cr) are presented in Figure 3a; the pore size distributions in these samples are shown in Figure 3b. Calculated parameters of porous structure are given in Table 1. According to the adsorption data, in pure MIL-101(Cr) the total pore volumes estimated using BET and DFT methods are 1.34 and 1.26 cm^3^/g, respectively.

The density of the guest-free MIL-101(Cr) estimated from crystallographic data [35] is 0.440 g/cm^3^ corresponding to the molar volume of 1553 cm^3^/mol or the specific volume of 2.273 cm^3^/g. The isotherm of nitrogen adsorption should be attributed to type IV(b) according to IUPAC classification, which is characteristic of mesoporous compounds with a narrow pore size distribution.

In the MIL-101(Cr) structure there are three types of cages with the diameters of 1.0, 1.8 and 3.2 nm, as evidenced from the data presented in Figure 3b. These values are slightly less than pore sizes values estimated from crystallographic data presented in Figure 1. It might be proposed that only pores with relatively large aperture can be filled by salt. The total volume of large pores with a radius of 0.9 and 1.6 nm is roughly estimated as 55 % of the total pore volume and is nearly 0.693 cm^3^/g (according to DFT calculations) corresponding to a pore volume per mole of MIL-101(Cr) equal to 474 cm^3^/mol. These data were used as initial parameters for further calculations.

Nitrogen adsorption isotherms for composites 0.887 LiClO_4_−0.113MIL-101(Cr) and 0.950 LiClO_4_−0.050 MIL-101(Cr) should be attributed to the type IV(b) which is typical for mesoporous samples, similarly to pure MIL-101(Cr). The total pore volume strongly decreases with the LiClO_4_ content. Moreover, the porous structure gradually changes with the increase in the concentration of the salt: (i) the mean diameter of the large pores decreases from 1.6 nm to 1.5 nm; (ii) the peak on the size distribution function at 0.9 nm decreases and splits into a wide maximum at 0.8 nm and a maximum at 1 nm; (iii) at high LiClO_4_ loading the peak at 0.5 nm splits, with two maxima at 0.45 nm and 0.56 nm. The observed changes in the porous structure may be caused by the insertion of LiClO_4_ into the cages of MIL-101(Cr) resulting in redistribution of the salt over the pore volume and partial pore blocking.

Adsorption isotherm for composites with the molar fraction of 0.008 and 0.017 MIL-101(Cr) should be attributed as Type III isotherm which is characteristic for non-porous materials. Evidently, in these samples practically all pores are filled or blocked by LiClO_4_ and the experimentally observed pore volume values are very low.

### 3.3. Transport Properties of Composites

Preliminary studies of ionic conductivity of LiClO_4_–MIL-101(Cr) composites were reported in paper [21], where the influence of humidity on the conductivity of the composites was studied in detail. It was also demonstrated that lithium cations are dominant charge carriers in the solid electrolytes under study. During the first heating, conductivity values of the composites are rather high and unstable in time, possibly due to strong adsorption of water from the gas phase. After prolonged heating at temperatures above 170 °C the conductivity values become reproducible in further heating-cooling cycles. Only these values will be discussed further. In this work we present the data not reported in paper [21] and discuss the conductivity data in more detail.

Temperature dependences of conductivity for composites LiClO_4_–MIL-101(Cr) are presented in Figure S1 (see Supplementary Materials). As seen, conductivity values obey the Arrhenius law *σ* = (A/T)·exp(−E_a_/RT), where A is the pre-exponential factor and E_a_ is the activation energy. Concentration dependences of conductivity *σ*(x) and the activation energy E_a_ for LiClO_4_-MIL-101(Cr) composites are shown in Figure S2 (see Supplementary Materials). The conductivity goes through the maximum as a function of the MIL-101(Cr) content in nanocomposite and reaches the maximum of *σ* = 7.4·10^−4^ S/cm at 150 °C at nearly 6 mole percent of MOF. The concentration dependence of conductivity with a maximum is typical for composite solid electrolytes [7,9] and is usually caused by the contribution of the interfaces to the overall conductivity of the composites.

The activation energy for conductivity decreases from 93.6 kJ/mol for pure lithium perchlorate to 52–58 kJ/mol for (1−x)LiClO_4_-xMIL-101(Cr) nanocomposites with x = 0.02–0.06 and increases with further increase in the concentration of MIL-101(Cr). Relatively low values of the activation energy are typical for LiClO_4_-based nanocomposites, such as LiClO_4_-Al_2_O_3_ [10,12], LiClO_4_-LiAlO_2_ [6], LiClO_4_-MgO [11], LiClO_4_-MCM-41 [14], in which the amorphous phase of the ionic salt is stabilized at LiClO_4_/oxide interfaces [9]. Possible reasons for the increase in the activation energy at high concentration of the MOF will be discussed below in the section devoted to the data interpretation.

## 4. Interpretation of the Data

### 4.1. The Model of the Composite Morphology

For quantitative interpretation of the textural and conductivity data one should consider a model of the composite. Evidently, the conductivity strongly depends on both the porous structure of MOF and the composite morphology. In general, a two-component composite comprises a mixture of the components and the character of mixing has strong influence on the morphology of the composite and, hence, on its properties. As a rule, composites are composed of the particles of individual components randomly distributed over the composite. The description of the properties of such systems is rather problematic due to the complicated character of the grain size distribution of both the components. The influence of interfaces makes the problem more complicated. In contrast, the properties of composites with components having well-defined morphology can be described more precisely in accordance with their composition and structure. The composites based on porous metal-organic frameworks are examples of such systems.

**Figure 4:**
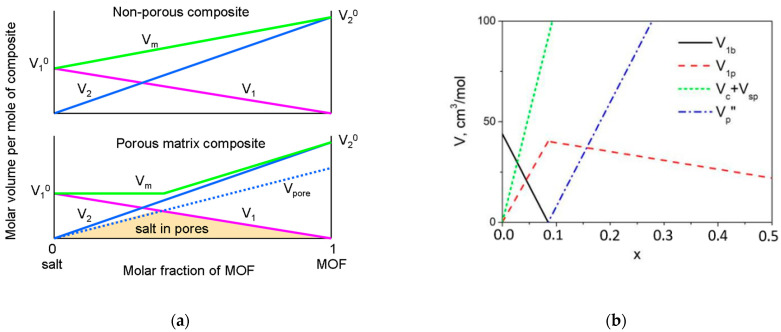
(**a**) The variation of molar volumes (per mole of the composite) with the molar fraction of the additive) for the composite (V_m_, green line), initial salt (V_1_, magenta line) and the additive (V_2_, blue line) which were taken for preparation of the composite in the case for non-porous additive (upper plot) and porous matrix composite (bottom plot). The dotted line corresponds to the pore volume (V_pores_); the colored region indicates the volume of the salt located in the pores. (**b**) Concentration dependences of the molar volumes (per mole of the mixture) of bulk lithium perchlorate (V_1b_), the lithium perchlorate located in the large pores (V_1p_), the cage MIL-101 together with small pores (V_c_ + V_sp_) and the volume of unfilled large pores or empty space in the large pores (V_p_″). The dependences were obtained from Equations (3)–(5) at *x* < *x*_max_ and Equations (7) and (8) at *x* > *x*_max_ using the values V_1_^0^ =44 cm^3^/mol; V_p_^0^ = 474 cm^3^/mol; V_c_^0^ = 713 cm^3^/mol; V_sp_^0^ = 387 cm^3^/mol, corresponding to *x*_max_ = 0.085.

Let us consider the change in the morphology of the composite MX-A consisting of the ionic salt MX and the porous chemically inert component A. At a low concentration of the component A, the volume of the composite may not increase with the content of A as would be expected from a simple mixing rule (Figure 4a). It is caused by the effect of the pore filling and the formation of a more dense system. The densification effect is maximal at a concentration of A corresponding to the complete filling of the pores when the MX volume is equal to the total volume of the pores. At a higher concentration of A, the pore volume exceeds the volume of MX. It may result in two possible variants of the composite morphology: random distribution of filled pores over the composite matrix, or uniform filling of all pores provided that MX covers the surface of each pore by a layer and the thickness of this layer decreases with the A content.

This model can be applied to LiClO_4_-MIL-101(Cr) composites. In Table A1 (see Appendix A), all designations of variables used in the formulas below and their meaning are summarized for clarity. First, one can plot the dependence of the total V_m_ and partial volumes V_1_ and V_2_ of the components, LiClO_4_ and MIL-101(Cr), respectively, as a function of the molar fraction of MIL-101(Cr) in the composites (1−x)LiClO_4_−xMIL-101(Cr), where x is the molar fraction. The values corresponding to x = 0 and x = 1 are equal to molar volumes of pure lithium perchlorate and MIL-101(Cr), *V*_1_^0^ and *V*_2_^0^, respectively. The value of the molar volume of lithium perchlorate located in the pores is assumed to be equal to *V*_1_^0^. The molar volume of pure MIL-101(Cr) could be presented as a sum of three terms: the volume of framework walls, *V_c_*^0^, the volume of microporous cages which are inaccessible to the ionic salt (small pores), *V_sp_*^0^, and the molar volume of mesocages accessible to the salt (large pores), *V_p_*^0^.

If both the components were non-porous, the dependence of *V_m_* on concentration for the composite would be linear:

(1) $$V_{m}=V_{1}+V_{2}=V_{1}^{0}\left(1-x\right)+\left(V_{c}^{0}+V_{sp}^{0}+V_{p}^{0}\right)x$$

The density of such composites may be estimated from the equation

(2) $$\rho =\frac{M}{V_{m}}=\frac{M_{1}\left(1-x\right)+M_{2}x}{V_{1}^{0}\left(1-x\right)+\left(V_{c}^{0}+V_{sp}^{0}+V_{p}^{0}\right)x}$$

where *M*, *M*_1_ and *M*_2_ are molecular weights of the composite, LiClO_4_ and MIL-101(Cr), respectively. As lithium perchlorate enters into the MIL-101(Cr) cage, simple linear dependence (1) and the relation (2) hold no more and the effect of the salt penetration should be taken into account. The total volume of lithium perchlorate, *V*_1_, is redistributed into the two parts: the volume of the bulk salt outside the pores, *V*_1*b*_, and the volume of the salt located inside the pores, V_1p_. In this case the volume of the pores accessible to the ionic salt, *V_p_*, is also distributed to two terms: the volume of the pores filled with the salt, *V_p_*′, and the volume of empty pores, *V_p_*″ (or the total volume of empty space in the pores).

At a low concentration of the MIL-101(Cr), the composite may be represented as a mixture of the bulk LiClO_4_ and the MIL-101(Cr) particles with pores completely filled with lithium perchlorate. It means that *V*_1*p*_ = *V_p_*′; *V_p_*″ = 0. Then, volumes *V*_1*b*_, *V*_1*p*_, *V_c_* and *V_p_*′ are given by the following expressions:

(3) $$V_{m}=V_{1}+V_{2}=V_{1}^{0}\left(1-x\right)+\left(V_{c}^{0}+V_{sp}^{0}+V_{p}^{0}\right)x$$

(4) $$V_{1b}=V_{1}^{0}\left(1-x\right)-V_{1p}=V_{1}^{0}\left(1-x\right)-V_{p}^{0}x$$

(5) $$V_{c}+V_{sp}=\left(V_{c}^{0}+V_{sp}^{0}\right)x$$

As usual, *V_p_*^0^ > *V*_1_^0^; therefore, at some concentration of MIL-101(Cr), the volume of the bulk LiClO_4_, V_1b_, decreases to zero and all lithium perchlorate gets into the pores. At this point, where *x = x*_max_, the volume of the salt included into the pores, *V*_1*p*_, goes through a maximum. The value of *x*_max_ may be found from Equation (4) at *V*_1*b*_ = 0 and is equal to

(6) $$x_{\max}=\frac{V_{1}^{0}}{V_{1}^{0}+V_{p}^{0}}$$

From the values *V*_1_^0^ = 43.96 cm^3^/mol (estimated from the values *M*_1_ = 106.39 g/mol and the density 2.42 g/cm^3^ [11]) and *V*_2_^0^ = 474 cm^3^/mol (estimated from the adsorption data presented in the Section 3.2) one finds that *x*_max_ = 0.085.

At *x* > *x*_max_, the composite comprises the MIL-101(Cr) framework and LiClO_4_ which is completely located in the pores. In this case *V*_1*b*_ = 0; *V_c_* is given by Equation (5) and *V*_1*p*_ may be found from a relation

(7) $$V_{1p}=V_{1}^{0}\left(1-x\right)$$

As was mentioned above, there are two variants of the composite morphology. In the case of random distribution of the completely filled pores

(8) $${V_{p}}^{\prime \prime }=V_{p}-V_{1p}=V_{p}^{0}x-V_{1}^{0}\left(1-x\right)$$

Equation (8) is valid also for another case when the salt is uniformly distributed over the surfaces of the pores and the thickness of the salt layer decreases with *x*. However, in this case the *V_p_*″ value has another physical meaning: it corresponds to the total volume of empty space in the pores. Theoretical concentration dependences of volumes of lithium perchlorate, cage and different pores of MIL-101(Cr) per mole of the composite are presented in Figure 4b.

### 4.2. Estimation of the Pore Volume in Composites: Comparison with Adsorption Data

The model may be applied for the description of experimental data obtained by nitrogen adsorption. Accordingly, the total volume of small pores, inaccessible to LiClO_4_, is given by the equation

(9) $$V_{sp}=V_{sp}^{0}x$$

whereas the volume of large pores at *x* < *x*_max_ is equal to zero and at *x* > *x*_max_ is given by Equation (8). The total pore volume measured by the nitrogen adsorption is equal to a sum

(10) $$V_{tot}=V_{sp}+{V_{p}}^{\prime \prime }$$

Values of the pore volume *V_tot_* (measured in cm^3^/mol) can be converted into the pore volume *V_pore_* values (measured in cm^3^/g) by a relation

(11) $$V_{tot}=V_{pore}\left[M_{1}\left(1-x\right)+M_{2}x\right]$$

**Figure 5:**
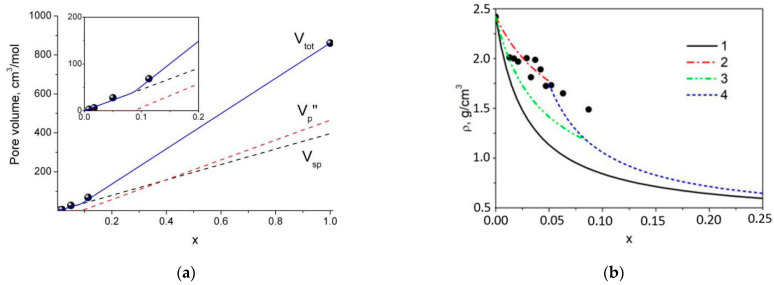
(**a**) Experimental values of the MIL-101(Cr) pore volume (symbols) in comparison with theoretical dependences obtained from Equations (8)–(10) for the total volume of unfilled pores (*V_tot_*), solid line, and values of *V_p_*″ and *V_sp_* (dash lines). The dependences were obtained at *V*_1_^0^ = 44 cm^3^/mol; *V_p_*^0^ = 474 cm^3^/mol; *V_sp_*^0^ = 387 cm^3^/mol. (**b**) Experimental values of density of the composite pellets (points) in comparison with theoretical dependences obtained using Equation (2) for a mixture of non-porous components (curve 1). Curves 2 and 3 were obtained for *x* < *x*_max_ from Equation (13) at *V_p_*^0^ = 861 cm^3^/mol and *V_p_*^0^ = 474 cm^3^/mol, respectively; curve 4 was obtained from Equation (14) for *x* > *x*_max_. In all the cases *V_c_*^0^ + *V_sp_*^0^ + *V_p_* = 1553 cm^3^/mol.

As seen from Figure 5a, a satisfactory agreement between experimental (obtained from the *V_pore_* data presented in Table 1) and theoretical *V_tot_* data may be observed at *V_sp_*^0^ = 387 cm^3^/mol, provided that *V*_1_^0^ = 44 cm^3^/mol and *V_p_*^0^ = 474 cm^3^/mol. The value of *x*_max_ = 0.085 can be estimated from these values.

### 4.3. Calculation of Composites’ Density

The density of the composites may be calculated as follows: At *x* < *x*_max_ the molar volume is given by a sum of Equations (4) and (5)

(12) $$V=V_{1}^{0}\left(1-x\right)+\left(V_{c}^{0}+V_{sp}^{0}\right)x$$

whereas at *x* > *x*_max_ the volume of the composite is completely defined by the sum of Equations (5) and (8). As a result, density of the composite will be equal to

(13) $$\rho =\frac{M_{1}\left(1-x\right)+M_{2}x}{V_{1}^{0}\left(1-x\right)+\left(V_{c}^{0}+V_{sp}^{0}\right)x}\;\mathrm{at}\;x\;<\;x_{\max}$$

(14) $$\rho =\frac{M_{1}\left(1-x\right)+M_{2}x}{\left(V_{c}^{0}+V_{sp}^{0}+V_{p}^{0}\right)x}\;\mathrm{at}\;x>\;x_{\max}$$

Comparison of Equations (13) and (14) with Equation (2) shows that the density of the composite with penetrable pores always exceeds the density of the composite with non-porous additives. Figure 5b shows experimental density data for pelleted samples of LiClO_4_–MIL-101(Cr) composites in comparison with theoretical dependences (2) and (10). The value of *x*_max_ can be found from the intersection of lines corresponding to the dependences (13) and (14). It is seen that the model taking into account the penetration of the salt in the pores satisfactorily describes experimental data. It should be also noted that the experimental data are more close to the theoretical curve 2 obtained at *V_p_*^0^ = 860 cm^3^/mol (for *x*_max_ = 0.05) and exceed the values found from the adsorption studies (*V_p_*^0^ = 474 cm^3^/mol, *x*_max_ = 0.085, curve 3). Hence, the volume of accessible pores in pellets seems to be higher than that in the powdered samples. These data may be useful in preparation of other functional composites [36,37].

### 4.4. Calculation of the Ionic Conductivity

For quantitative interpretation of the conductivity data, one can use the general mixing equation proposed earlier for estimation of the transport properties of two-phase conductors [29]. This equation has a rather simple form compared to effective medium and percolation approaches and fairly describes conductivity of composites with different morphology, including ones characterized by percolation-type conductivity behavior in a wide composition range. The equation has the form

(15) $$\sigma^{\alpha_{1}\left(1-f\right)+\alpha_{2}f}=\sigma_{1}^{\alpha_{1}\left(1-f\right)+\alpha_{2}f}\left(1-f\right)+\sigma_{2}^{\alpha_{1}\left(1-f\right)+\alpha_{2}f}f$$

where *f* is the volume fraction of the component 2; *σ*_1_ and *σ*_2_ are conductivities of pure phases 1 and 2; parameters *α*_1_ and *α*_2_ are defined by the composite morphology in the dilute limits *f* → 0 and *f* → 1, respectively; −1 ≤ *α*_1_, *α*_2_ ≤ 1. The case of *α*_1_ = 1, *α*_2_ = 1 corresponds to parallel arrangements of the phases in the composite when *σ* = *σ*_1_ (1 − *f*) + *σ*_2_·f; when *α*_1_ = −1, *α*_2_ = −1 the components are arranged perpendicular to the electric field and total conductivity is equal to *σ*^−1^ = *σ*_1_^−1^ (1 − *f*) + *σ*_2_^−1^ ·f. The classical equation for the case of spherical isolated particles of phase 2 randomly distributed over conducting phase 1 corresponds to the value of *α*_1_ = 2/3 and for the case when conducting particles are located in dielectric media *α*_2_ = −1/3. The exponent in Equation (15) changes with the volume fraction taking into account gradual transformation of the morphology of the composite as a function of the concentration. It allows one to describe a percolation transition from conductive to dielectric behavior of the composite. Earlier, Equation (15) was used for description of the conductivity of composite solid electrolytes with different morphologies, such as a random mixture and the ceramics in which the ionic conducting phase is located on grain boundaries [30]. In the present work we apply the model for description of conductivity in the porous matrix of MIL-101(Cr) taking into account the filling of the pore with the lithium salt.

Conductivity of LiClO_4_−MIL-101(Cr) composites goes through a maximum at a concentration close to theoretical value of *x*_max_, corresponding to the maximum value of *V*_1*p*_. This suggests that the enhanced conductivity is caused by lithium perchlorate located in the pores of MIL. At *x* = *x*_max_, all ionic salt is within the pores and this value separates two concentration regions:

1. At *x* < *x*_max_ the composite may be regarded as a two-phase mixture of bulk LiClO_4_ and the MOF cage with the pores completely filled with LiClO_4_. It can be shown that relative volume fractions of the bulk LiClO_4_ and MIL-101(Cr) with filled pores, *φ*_1_ and *φ*_2_, respectively, are given by relations:

(16) $$\varphi_{1}=\frac{V_{1}^{0}\left(1-x\right)-V_{p}^{0}x}{V_{1}^{0}\left(1-x\right)+\left(V_{c}^{0}+V_{sp}^{0}\right)x}=\frac{x_{\max}-x}{x_{\max}}\cdot \frac{1}{1-\left(1-\theta \right)x}$$

(17) $$\varphi_{2}=\frac{\left(V_{c}^{0}+V_{sp}^{0}+V_{p}^{0}\right)x}{V_{1}^{0}\left(1-x\right)+\left(V_{c}^{0}+V_{sp}^{0}\right)x}=\frac{x}{x_{\max}}\cdot \frac{1-\left(1-\theta \right)x_{\max}}{1-\left(1-\theta \right)x}$$

where *θ* = (*V_c_*^0^ + *V_sp_*^0^)/*V*_1_^0^. It is seen that *φ*_1_ + *φ*_2_ = 1 in the concentration range 0 ≤ *x* ≤ *x*_max_. At *x* = *x*_max_ the fraction of accessible pores filled with the ionic salt is equal to 1. The mixing equation, Equation (15), for this region may be written in the form:

(18) $$\sigma^{\alpha_{1}\left(1-\varphi_{2}\right)+\alpha_{2}\varphi_{2}}=\sigma_{1}^{\alpha_{1}\left(1-\varphi_{2}\right)+\alpha_{2}\varphi_{2}}\left(1-\varphi_{2}\right)+\sigma_{1p}^{\alpha_{1}\left(1-\varphi_{2}\right)+\alpha_{2}\varphi_{2}}\varphi_{2}$$

where *σ*_1_ is the bulk conductivity of pure lithium perchlorate; parameters *α*_1_ and *α*_2_ depend on the composite morphology and in first approximation one can use their theoretical values for random distribution of components: *α*_1_ = −1/3 and *α*_2_ = 2/3; *σ*_1*p*_ is the conductivity of LiClO_4_ located in the pores which is much higher than *σ*_1_ due to the highly disordered amorphous structure of the ionic salt in the pores. At *x* = *x*_max_ the concentration of the amorphous phase reaches a maximum corresponding to the highest conductivity equal to *σ*_1*p*_.

2. At *x* > *x*_max_ the composite may be considered as the mixture of lithium perchlorate located in the pores and the cage of MIL-101(Cr), including the empty space in the pores or unfilled pores. Relative volume fractions of such components are given by relations:

(19) $$\phi_{1}=\frac{\left(V_{1}^{0}+V_{c}^{*}\right)\left(1-x\right)}{\left(V_{c}^{0}+V_{sp}^{0}+V_{p}^{0}\right)x}=\frac{1-x}{1-x_{\max}}\cdot \frac{x_{m}}{x}$$

(20) $$\phi_{2}=\frac{\left(V_{c}^{0}+V_{sp}^{0}+V_{p}^{0}\right)x-\left(V_{1}^{0}+V_{c}^{*}\right)\left(1-x\right)}{\left(V_{c}^{0}+V_{sp}^{0}+V_{p}^{0}\right)x}=\frac{x-x_{\max}}{1-x_{\max}}\cdot \frac{1}{x}$$

where V_c_* = *V*_1_^0^·(*V_c_*^0^ + *V_sp_*^0^)/*V_p_*^0^. In the concentration range *x*_max_ ≤ *x* ≤ 1 the volume of filled pores decreases and the volume of empty space in the cage increases; *ϕ*_1_ + *ϕ*_2_ = 1. In this case, the mixing equation has the form

(21) $$\sigma^{\beta_{1}\left(1-\phi_{2}\right)+\beta_{2}\phi_{2}}=\sigma_{1p}^{\beta_{1}\left(1-\phi_{2}\right)+\beta_{2}\phi_{2}}\left(1-\phi_{2}\right)+\sigma_{2}^{\beta_{1}\left(1-\phi_{2}\right)+\beta_{2}\phi_{2}}\phi_{2}$$

where *σ*_1p_ is the same value as in Equation (18); *σ*_2_ is the conductivity of pure MIL-101(Cr); parameters *β*_1_ and *β*_2_ (−1 ≤ *β*_1_, *β*_2_ ≤ 1) are defined by the distribution of the components over the porous matrix of MIL-101(Cr).

**Figure 6:**
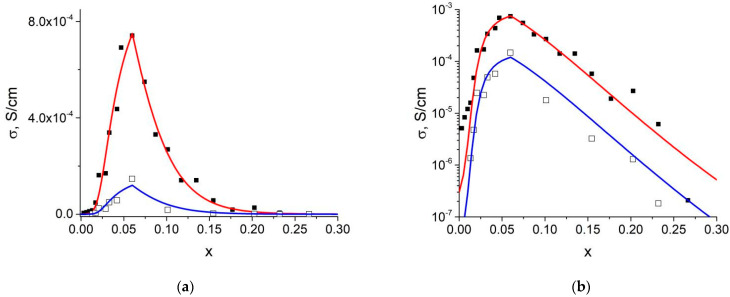
Concentration dependence of conductivity for the (1−x)LiClO_4_–xMIL-101 composites presented in linear (**a**) and logarithmic scale (**b**). Points are experimental values, solid lines are theoretical curves obtained in frames of the proposed model using Equations (18) and (21) for *x*_max_ = 0.06; *α*_1_ = −0.33; *α*_2_ = 0.67; *β*_1_ = 0.80; *β*_2_ = 0.15 and the conductivity values: *σ*_1_ = 3.10^−7^ S/cm; *σ*_1p_ = 7.4·10^−4^ S/cm; *σ*_2_ = 1.10^−11^ S/cm (for 150 °C, filled symbols, red lines) and *σ*_1_ = 3.10^−8^ S/cm; *σ*_1p_ = 1.2·10^−4^ S/cm; *σ*_2_ = 1.10^−11^ S/cm (for 100 °C, open symbols, blue lines).

Figure 6 shows theoretical *σ*(*x*) curves obtained from Equations (18) and (21) in comparison with experimental data. The best fit was obtained for *x*_max_ ≈ 0.06. According to Equation (6), this value corresponds to the volume of filled pores equal to *V*_2_^0^ ≈ 689 cm^3^/mol, corresponding to the total volume of filled pores of 1.01 cm^3^/g. This value is 80% of the total pore volume, 1.26 cm^3^/g, determined from nitrogen adsorption isotherms by the DFT method (see Table 1). Therefore, in contrast to the composites investigated by nitrogen adsorption technique (in which only large pores were filled by ionic salt), in samples used for conductivity measurements both large and small pores were practically completely filled with lithium perchlorate due to compaction of the pellets followed by a prolonged heating at elevated temperatures. As a result, the value of *x*_max_ determined from adsorption data (*x*_max_ = 0.085) becomes higher than the value *x*_max_ = 0.06 estimated from analysis of the conductivity data. A similar effect was observed for the density of composite pellets (see Figure 5b).

At *x* < *x*_max_ theoretical dependence *σ*(*x*) is fairly described by Equation (18) with the values of *α*_1_ = −1/3 and *α*_2_ = 2/3 corresponding to a random mixing of LiClO_4_ and MIL-101 particles at low concentration of the MOF additive. Therefore, the composite indeed may be represented as a random mixture of MOF with completely filled pores and the bulk lithium perchlorate. With the increase of the concentration of LiClO_4_ located in the pores, conductivity increases and the activation energy falls to a low value typical for ionic conductivity of the amorphous phase of LiClO_4_ located in the pores.

At *x* > *x*_max_ the situation seems to be more complicated. The Equation (21) allows one to fit the experimental data; however, both the values of *β*_1_ = 0.80 and *β*_2_ = 0.15 are not typical for random mixtures indicating to a non-random distribution of LiClO_4_ over the pores of MIL-101(Cr).

It should be noted that the character of the concentration dependence of conductivity does not change with temperature. As seen from Figure 6, theoretical dependences *σ*(*x*) obtained using the same parameters *x*_max_, *α*_1_, *α*_2_, *β*_1_ and *β*_2_ satisfactorily fit experimental data for both temperatures, 150 and 100 °C. Constancy of the *x*_max_, *α*_1_, *α*_2_, *β*_1_ and *β*_2_ values indicates that the morphology of the composites does not change with temperature.

Assuming that LiClO_4_ covers uniformly the surface of pores and the accessible pores have spherical shape, one can estimate the *ϕ*_2_ value using a relation *ϕ*_2_ = (1 − *λ*/*d_p_*)^1/3^, where *λ* and *d_p_* are the thickness of the salt layer and the pore size, respectively. From the value of *d_p_* = 3.2 nm, one can estimate the thickness of LiClO_4_ layer inside the pore as a function of *ϕ*_2_:

(22) $$\lambda =d_{p}\left(1-\sqrt[{3}]{\phi_{2}}\right)$$

Estimation shows that at *ϕ*_2_ = 0.5 (corresponding to *x* = 0.15) the thickness of the salt layer is nearly 0.64 nm, whereas an effective size of a single molecule of LiClO_4_ estimated from the molar volume of crystalline lithium perchlorate as *d*_1_ = (*V*_1_^0^/N_a_)^1/3^, where N_a_ is the Avogadro number, is equal to 0.41 nm. It means that at concentrations *x* > 0.15 the pores are covered on average by a monomolecular layer of lithium perchlorate. Possibly, a continuous decrease in the thickness of the salt layer covering inner surfaces of pores leads to an increase in the activation energy of conductivity E_a_ as a function of the concentration (see Figure S2b, Supplementary Materials). In this case, the *σ*_1*p*_ value should depend on the concentration of MOF; for correct fitting of the conductivity data used in Equation (21), one should have information on the concentration dependence of *σ*_1*p*_ in the composite.

## 5. Conclusions

Composite solid electrolytes with high lithium-ion conductivity are key materials for solid-state lithium batteries. In this regard, MIL-101(Cr) is an excellent porous matrix for preparation of nanocomposites with unusual physical and chemical properties.

It was shown by methods of X-ray diffraction, nitrogen adsorption and conductivity measurements, that lithium perchlorate easily penetrates into the pores of MIL-101(Cr) with formation of nanocomposites. Ionic salt in the pores occurs in the amorphous state. According to the results of adsorption studies, only part of the total pore volume is occupied by the lithium salt, as the smaller pores with the average size of nearly 0.5 nm are not accessible to the salt. The composites have a high ionic conductivity. The concentration dependence of the conductivity of (1−x)LiClO_4_–xMIL-101(Cr) composites has a maximum at *x* = *x*_max_ = 0.06 (*σ* = 7.4·10^−4^ S/cm and *σ* = 1.2·10^−4^ S/cm at 150 and 100 °C, respectively). The activation energy of conductivity decreases with x from 93.6 kJ/mol for pure LiClO_4_ to 58 kJ/mol at *x* = *x*_max_ and *x* > *x*_max_ monotonically increases with *x*.

The experimental values of the pore volume, pellets density and ionic conductivity of LiClO_4_–MIL-101(Cr) composites were quantitatively interpreted in terms of the pore filling model of the nanocomposite according to which the overall concentration range may be separated in two regions: at *x* < *x*_max_ the composites consist of bulk LiClO_4_ and the MOF in which all accessible pores are filled with the lithium perchlorate; at *x* = *x*_max_ all accessible pores are completely filled by the salt and the concentration of bulk LiClO_4_ falls to zero; at *x* > *x*_max_ all lithium perchlorate is located in the accessible pores and only part of the pore volume is filled with the salt. The model satisfactorily describes experimental data. It turned that the *x*_max_ value determined from the adsorption experiments (*x*_max_ = 0.085) exceeds the corresponding value found from conductivity data (*x*_max_ = 0.06). The difference might be caused by filling the small pores during pressing the pellets and prolonged heating of the composite at elevated temperature prior to conductivity measurements. Analysis of the conductivity data suggests that at *x* > *x*_max_ lithium perchlorate forms layers inside the pores. The decrease in the layer thickness with x leads to the increase in the activation energy of conductivity of the composites.

As a result of this study, it may be concluded that nanocomposites LiClO_4_–MIL-101(Cr) have high lithium ion conductivity and may be used for development of solid state electrochemical devices. The proposed model of the composite structure can be useful for the quantitative description of experimental data on the conductivity of solid electrolytes based on porous additives with ordered pores, including various types of MOFs.

## Figures and Tables

**Table 1:** The porous structure parameters of LiClO_4_-MIL-101(Cr) composites.

Composition	Specific Surface Area/m^2^·g^−1^		V_pore_/cm^3^·g^−1^		V_ads(N2)_ */cm^3^·g^−1^
	BET	DFT	Total *	DFT	
0.992LiClO_4_–0.008MIL-101(Cr)	39	25.6	0.028	0.028	18.1
0.983LiClO_4_–0.017MIL-101(Cr)	76	56.7	0.056	0.054	36.2
0.950LiClO_4_–0.050MIL-101(Cr)	413	265.2	0.212	0.205	137.3
0.887LiClO_4_–0.113MIL-101(Cr)	888	546.1	0.423	0.399	273.6
MIL-101(Cr)	2200	1776	1.34	1.26	889

* measured at P/P_0_ = 0.95.

## Data Availability

The data presented in this study are available on request from the corresponding author.

## Supplementary Materials

The following supporting information can be downloaded at: https://www.mdpi.com/article/10.3390/nano12193263/s1, Figure S1: Temperature dependences of conductivity for the (1−x)LiClO_4_−xMIL-101(Cr) composites with small (a) and high (b) concentration of the porous additive. Figure S2: Concentration dependences of conductivity at 150 °C (a) and the activation energy of conductivity (b) for the (1−x)LiClO_4−_xMIL-101(Cr) composites.

## Appendix A

**Table A1:** List of variables and designations used in the text.

Parameter	Description
x	Molar fraction of MIL-101(Cr)
M, M_1_ and M_2_	Molecular mass of the composite (M), the first component, LiClO_4_, and the second component MIL-101(Cr) in composite, respectively [g/mol]
V_m_	Total molar volume of composite [cm^3^/mol]
V_1_	The effective partial molar volume of the 1-st component (LiClO_4_) [cm^3^/mol]
V_2_	The effective partial molar volume of the 2-nd component (MIL-101(Cr)) in composite [cm^3^/mol]
V_1_^0^	The molar volume of the 1-st component, the pristine LiClO_4_ [cm^3^/mol]
V_2_^0^	The molar volume of the 2-nd component, the pristine MIL-101(Cr) [cm^3^/mol]
V_c_^0^	The molar volume of the framework walls in pristine MIL-101(Cr) [cm^3^/mol]
V_sp_^0^	The molar volume of microporous cages (small pores) inaccessible to the salt in pristine MIL-101(Cr) [cm^3^/mol]
V_p_^0^	The molar volume of mesocages accessible to the salt in pristine MIL-101(Cr) [cm^3^/mol]
V_c_	The volume of the framework walls of MIL-101(Cr) [cm^3^/mol] per mole of the composite
V_sp_	The volume of microporous cages (small pores) in MIL-101(Cr), which are inaccessible to the salt, per mole of the composite [cm^3^/mol]
V_p_	The volume of mesocages in MIL-101(Cr) accessible to the salt in composite per mole of the composite [cm^3^/mol]
V_1p_	The volume of the salt located inside the (accessible) pores of MIL-101(Cr) per mole of the composite [cm^3^/mol]
V_1b_	The volume of the salt located in the bulk state outside the pores of MIL-101(Cr) per mole of the composite [cm^3^/mol]
V_p_′	The volume of mesocages filled with the salt per mole of the composite, [cm^3^/mol]
V_p_″	The volume of empty mesocages per mole of the composite [cm^3^/mol]
V_tot_	The total molar volume of pores in MIL-101(Cr) [cm^3^/mol]
V_pore_	The total volume of pores in MIL-101(Cr) [cm^3^/g]
x_max_	The molar fraction of MIL-101(Cr) at which the volume of the ionic salt located in the pores reaches a maximum value corresponding to the volume of accessible pores
*ρ*	The density of the composite [g/cm^3^]
σ, A, E_a_	The conductivity of the composite [S/cm], the pre-exponential factor [S·K/cm] and the activation energy [kJ/mol] for conductivity of composite, respectively.
σ_1_, σ_2_	Conductivities of the components 1 and 2 in Equation (15); conductivities [S/cm] of pure LiClO_4_ and MIL-101(Cr) in Equations (18) and (21), respectively.
f	The volume fraction of the component 2 in the mixing Equation (15)
σ_1p_	Conductivity of LiClO_4_ located in the pores of MIL-101(Cr) [S/cm].
α_1_, α_2_	Parameters of the mixing equations, Equations (15) and (18); −1 ≤ α_1_, α_2_ ≤ 1
φ_1,_ φ_2_	Relative volume fractions of the components used in Equations (16)–(18)
ϕ_1_, ϕ_2_ β_1_, β_2_	Relative volume fractions of the components used in Equations (19)–(21) Parameters of the mixing equation, Equation (21); −1 ≤ β_1_, β_2_ ≤ 1
V_c_*	The parameter of Equations (19) and (20) defined by a relation: V_c_* = V_1_^0^·(V_c_^0^ + V_sp_^0^)/V_p_^0^
