# Supplementary material for: Effect of Pore Filling on Properties of Nanocomposites LiClO4–MIL–101(Cr) with High Ionic Conductivity
Source: Nanomaterials (Basel). 2022 Sep 20;12(19):3263. doi: 10.3390/nano12193263 (PMC9565636; doi:10.3390/nano12193263)
Supplement: Supplementary file 1 [file nanomaterials-12-03263-s001.zip › nanomaterials-1916643-supplementary.pdf]

nanomaterials

MDPI

Supplementary Materials

Effect of Pore Filling on Properties of Nanocomposites LiClO4–MIL–101(Cr) with High Ionic Conductivity

Nikolai Uvarov [1,*], Artem Ulihin [1], Valentina Ponomareva [1], Konstantin Kovalenko [2] and Vladimir Fedin [2]

[1] Institute of Solid State Chemistry and Mechanochemistry SB RAS, Kutateladze 18, Novosibirsk 630090, Russia; ulikhin@solid.nsc.ru (A.U.); ponomareva@solid.nsc.ru (V.P.)
[2] Nikolayev Institute of Inorganic Chemistry, SB RAS, Acad. Lavrentiev Ave. 3, Novosibirsk 630090, Russia; k.a.kovalenko@niic.nsc.ru (K.K.); cluster@niic.nsc.ru (V.F.)
* Correspondence: uvarov@solid.nsc.ru; Tel.: +7-383-233-2410

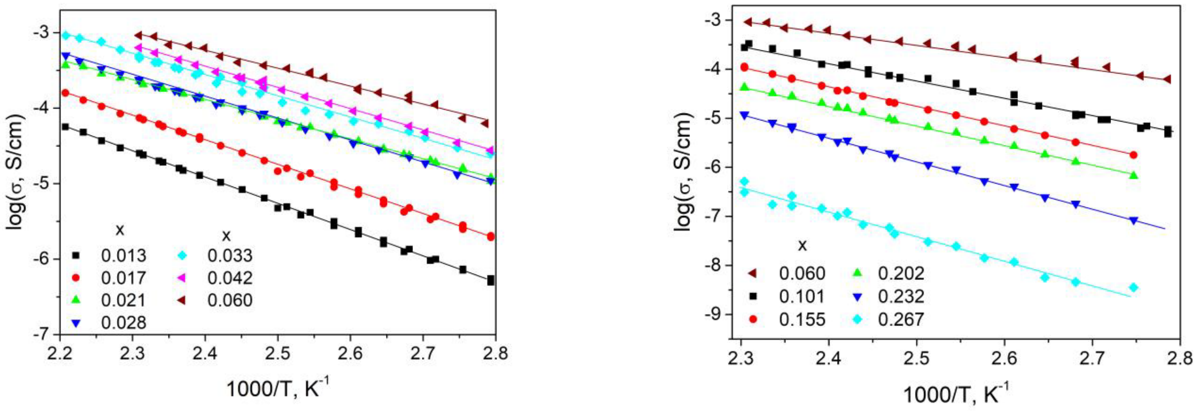

(**a**) (**b**)

Figure S1. Temperature dependences of conductivity for the (1−x)$LiClO_4$–xMIL-101(Cr) composites with small **(a)** and high **(b)** concentration of the porous additive.

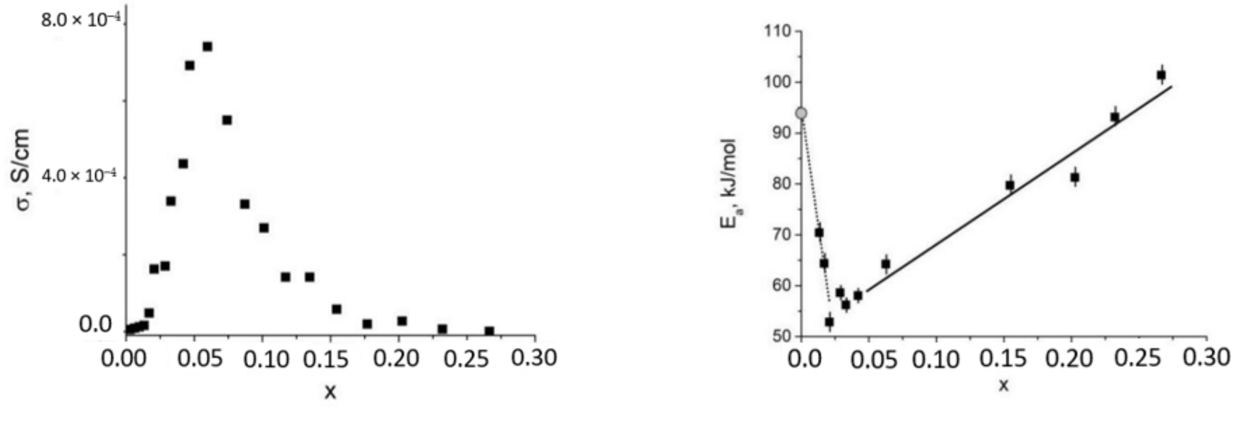

(**a**) (**b**)

Figure S2. Concentration dependences of conductivity at 150 °C (**a**) and the activation energy of conductivity (**b**) for the (1−x)$LiClO_4$-xMIL-101(Cr) composites.
